# Landscape of the metaplasmidome of deep-sea hydrothermal vents located at Arctic Mid-Ocean Ridges in the Norwegian–Greenland Sea: ecological insights from comparative analysis of plasmid identification tools

**DOI:** 10.1093/femsec/fiae124

**Published:** 2024-09-13

**Authors:** Karol Ciuchcinski, Runar Stokke, Ida Helene Steen, Lukasz Dziewit

**Affiliations:** Department of Environmental Microbiology and Biotechnology, Institute of Microbiology, Faculty of Biology, University of Warsaw,00-927, Warsaw, Poland; Department of Biological Sciences, Center for Deep Sea Research, University of Bergen, N-5020, Bergen, Norway; Department of Biological Sciences, Center for Deep Sea Research, University of Bergen, N-5020, Bergen, Norway; Department of Environmental Microbiology and Biotechnology, Institute of Microbiology, Faculty of Biology, University of Warsaw,00-927, Warsaw, Poland

**Keywords:** Arctic deep-sea hydrothermal vent, metaplasmidome, plasmid, plasmid classification, plasmid identification, thermophile

## Abstract

Plasmids are one of the key drivers of microbial adaptation and evolution. However, their diversity and role in adaptation, especially in extreme environments, remains largely unexplored. In this study, we aimed to identify, characterize, and compare plasmid sequences originating from samples collected from deep-sea hydrothermal vents located in Arctic Mid-Ocean Ridges. To achieve this, we employed, and benchmarked three recently developed plasmid identification tools—PlasX, GeNomad, and PLASMe—on metagenomic data from this unique ecosystem. To date, this is the first direct comparison of these computational methods in the context of data from extreme environments. Upon recovery of plasmid contigs, we performed a multiapproach analysis, focusing on identifying taxonomic and functional biases within datasets originating from each tool. Next, we implemented a majority voting system to identify high-confidence plasmid contigs, enhancing the reliability of our findings. By analysing the consensus plasmid sequences, we gained insights into their diversity, ecological roles, and adaptive significance. Within the high-confidence sequences, we identified a high abundance of *Pseudomonadota* and *Campylobacterota*, as well as multiple toxin–antitoxin systems. Our findings ensure a deeper understanding of how plasmids contribute to shaping microbial communities living under extreme conditions of hydrothermal vents, potentially uncovering novel adaptive mechanisms.

## Teaser

Benchmarking various bioinformatic tools uncovers novel plasmids from Arctic Mid-Ocean Ridge hydrothermal vents, shedding light on their ecological role in deep-sea microbial communities.

## Introduction

Plasmids, small circular DNA molecules capable of horizontal gene transfer, are key drivers of microbial adaptation and evolution (Ochman et al. 2000, Tokuda and Shintani 2024). While most often associated with the spread of antibiotic resistance, their broader ecological roles are increasingly recognized, and they include conferring adaptive features (also novel metabolic properties), resistance to metals and host–microbe interactions (Galetti et al. 2019, Alav and Buckner 2023, Gomathinayagam and Kodiveri Muthukaliannan 2024). Most research to date has focused on plasmids in mesophilic organisms, leaving these in extreme environments significantly understudied. This bias is evident in major databases, such as plasmid database PLSDB, where over 50% of the nearly 60 000 deposited sequences originate from just five genera (*Escherichia, Klebsiella, Enterococcus, Salmonella*, and *Staphylococcus*) (version 2023_11_03_v2) (Schmartz et al. 2022). In contrast, a focused review of literature and databases identified only 174 and 526 plasmid sequences from thermophiles and psychrophiles, respectively, highlighting the need for expanded research efforts in these understudied groups.

Deep-sea hydrothermal vents (DSHVs) represent a unique extreme habitat. Characterized by minimal light, high pressure, temperatures ranging from 2°C to 350°C, as well as highly variable chemical compositions and energy sources (Haase et al. 1995, Zeng et al. 2021). Compared to other extreme environments, such as polar regions, deserts, salt pans, or hot springs, deep-sea environments are also much more isolated (Mullineaux et al. 2018). While the influx and efflux of genetic information in DSHVs is limited, plasmids have been isolated from those remote communities (Lossouarn et al. 2015). In general, plasmids found in extreme environments tend to carry genes that directly benefit the survival of their host in given environments. For example, plasmids identified from polar environments can carry genes responsible for resistance to cold and UV radiation, as well as heavy metals and other toxic compounds, which pose the greatest threat in this environment (Dziewit and Bartosik 2014, Ciok et al. 2018, Makowska-Zawierucha et al. 2024). Similarly, plasmids isolated from DSHVs may carry genes encoding enzymes which could be attributed to adaptation to DNA damage at high temperatures (Makarova et al. 2002, Majerník et al. 2004, Lossouarn et al. 2015). However, due to the scarcity of reference data, many plasmid-borne genes, especially originating from extreme habitats, still remain poorly annotated, hindering a comprehensive understanding of their ecological roles.

Studying DSHV ecosystems is inherently challenging due to difficulties in sample collection and the inability to cultivate many microorganisms under laboratory conditions (Martiny 2019, Schultz et al. 2023). Since this makes it difficult to obtain plasmid DNA directly from environmental samples, alternative, enrichment-based methods have been proposed. In one of them, microbes coming from environmental samples are used to inoculate selective media, and the resulting cultures can be used to isolate plasmid DNA (Gorecki et al. 2021). Another method, which was employed to collect samples analysed in this study, utilizes *in situ* enrichment using cultivation chambers (Kaeberlein et al. 2002, Bollmann et al. 2007). While effective, it is important to remember that these methods introduce a “planned bias,” since only a relatively small portion of environmental microbes may be successfully cultivated (Dziurzynski et al. 2023).

In order to reduce this bias, many recent studies have employed shotgun metagenomics, which, in theory, should provide unbiased information about all environmental DNA found within a given sample (Hedlund et al. 2014, Gómez-Silva et al. 2019). Understandably, this approach introduces its own challenges. Most notably, recovery of plasmids from WGS sequencing data is especially difficult, given their smaller length and abundance (Fritz et al. 2019, Mendes et al. 2023). This problem is exacerbated even further for plasmids coming from marine environments (Meyer et al. 2022).

Addressing the challenges of plasmid identification from metagenomic data, recent advances in computational biology and artificial intelligence have led to development of several novel plasmid identification tools. In the span of last year, three new plasmid classifiers have been introduced, each promising improved accuracy and precision. PlasX, created by Yu et al. (2023), utilizes a logistic regression model trained on a large-scale dataset, providing improved recall and precision. GeNomad, a hybrid pipeline by Camargo et al. (2023), combines nucleotide sequence classification using an IGLOO-based encoder with custom marker gene identification, outperforming many existing tools in their benchmarks (Sourkov 2020, Camargo et al. 2023). Notably, PlasX was the second-best tool in almost all benchmarks presented in the GeNomad paper. Lastly, PLASMe, introduced by Tang et al. (2023), leverages a natural language processing-inspired approach, treating protein sequences as vocabulary for transformer models tailored to specific bacterial orders. To date, a direct comparison of these tools has not been conducted.

In this study, we aim to identify, characterize, and compare plasmid sequences derived from 14 environmental samples collected from DSHVs located in the Arctic Mid-Ocean Ridges (AMOR). By benchmarking the performance of PlasX, GeNomad, and PLASMe on this unique dataset, we aim to evaluate their biases and effectiveness in identifying plasmids from a complex, understudied environment. Furthermore, we seek to gain insights into the diversity, ecological roles, and adaptive significance of plasmids in these extremophilic microbial communities, contributing to a deeper understanding of their contributions to ecosystem function and evolution.

## Materials and methods

### Sample collection and processing, DNA extraction, and sequencing

A total of 14 samples were collected from hydrothermal vents at AMOR, located in the Norwegian–Greenland Sea (Table S1). Four were from the Loki’s Castle Vent Field, one from the Soria Moria vent field, and nine from *in situ* enrichments at the Bruse vent field (Stokke et al. 2020, Vulcano et al. 2022). Following the sampling, metagenomic DNA was isolated and sequenced as previously described (Stokke et al. 2020, Vulcano et al. 2022). Sequencing was performed in two batches, using the Illumina MiSeq (300 bp; samples M1-5, M10-14) and NovaSeq (150 bp; M19-21, M34) platforms.

### Bioinformatic analysis

#### Quality control and assembly

Following the sequencing, obtained data was processed and assembled using either Qiagen CLC Genomics Workbench (v11; MiSeq samples) or fastp (v 0.23.2) (Chen et al. 2018), with MEGAHIT v1.2.9 (Li et al. 2016). Only contigs over 500 bp were considered for further analysis.

#### Plasmid identification

Following assembly, contigs longer than 500 bp were used as input for three different plasmid identification tools. First, GeNomad v1.7.4 was used in end-to-end mode with the following flags: *–enable-score-calibration –disable-find-proviruses –cleanup*. Each task was given 40 CPU threads and 60 Gb of RAM (Camargo et al. 2023). Next, data for the PlasX pipeline were preprocessed by anvi'o, and plasmid contigs were identified by running the *search_de_novo_families* and *predict* commands with default parameters (Eren et al. 2021, Yu et al. 2023). Similarly, the tasks were assigned with the same computational resources. Finally, PLASMe was used with the unified transformer (*-u True*) (Tang et al. 2023). After prediction, results from all three tools were filtered based on score assigned to each contig. Only contigs with score ≥0.7 (where 0 is a chromosome and 1 is a plasmid) were marked as plasmid contigs.

#### Majority voting system for plasmid contigs selection

The set containing plasmid contigs remaining after filtering was then further refined using a majority voting system. Intersections between results of each tool were calculated via a custom Python script. Singletons (i.e. contigs only found in output of one tool) were classified as unlikely to be plasmids, and contigs found in intersections of two or all three tools were designated as high-confidence plasmid contigs.

### Characterization of identified plasmid contigs

Characterization was performed for all potential plasmid contigs, i.e both singletons and contigs from set intersections. All analyses were performed for contigs longer than 500 bp, except for taxonomic classification with Kraken2, which was performed using all contigs.

#### Taxonomic annotation

Taxonomy was assigned to predicted plasmid contigs using two approaches. First, the contigs were annotated using kraken2 (v.2.1.3) with Standard database (rev. 2023_04_13, obtained from https://benlangmead.github.io/aws-indexes/k2), using the –report-minimizer-data and –minimum-hit-groups 3 flags, as recommended by Lu et al. (2022) and Wood et al. (2019). Next, the contigs were annotated using MMseqs2 (v. 92d8cc375ea4cc4784e17150d10e0f9dc8004491) easy-taxonomy workflow (Steinegger and Söding 2017). The reference database used was the NCBI NR database (rev. 2023_02_20), obtained using the MMseqs2 *databases* workflow. Both tools were assigned 40 CPU cores and 120 Gb of RAM per task.

#### Functional annotation

Functional annotation of genes found on predicted plasmid contigs was performed using the eggNOG-mapper v2 suite (v2.1.12), utilizing Prodigal for gene calling and DIAMOND for protein alignment (Hyatt et al. 2010, Huerta-Cepas et al. 2019, Buchfink et al. 2021, Cantalapiedra et al. 2021). The emapper command was run with default parameters, using the contigs as an input (–itype metagenome).

#### Identification of genes with adaptation value

In order to identify genes carrying adaptive function, coding sequences (CDS) within each plasmid contig were predicted using Pyrodigal (v3.3.0)—a Python library binding to Prodigal (Hyatt et al. 2010, Larralde 2022).

Genes with adaptive value were identified from the results obtained from the eggNOG-mapper tool. The results were parsed, looking for genes assigned a specific KO number, gene name, GO term, as well as via text search within function descriptions. All filtering steps were performed using custom-made Python scripts.

#### Identification and description of mobilization for conjugal transfer, replication, and mating pair formation proteins

Identification and classification of mobilization for conjugal transfer (MOB), replication (REP), and mating pair formation (MPF) proteins within plasmid contigs was performed using MOB, REP, and MPF protein databases from the MOB-suite utility (Robertson and Nash 2018). Those databases were used to create diamond databases (*makedb*), and to search the genes of plasmid contigs using *diamond blastp*. Output data was then filtered using custom Python scripts, selecting hits with at least 50% sequence identity and 70% bidirectional coverage (*pident qcovhsp* and *qcovhsp* from *–outfmt 6* accordingly). Afterwards, only one database hit per protein (with lowest e-value) was selected.

#### Identification of ncRNA

Identification of ncRNA’s was performed using the Rfam database v14.10 and Infernal v 1.1.5 (Nawrocki and Eddy 2013, Kalvari et al. 2017). First, the covariance model database was created from the Rfam source files using *cmpress*. Next, *cmscan* was used to identify RNA sequences within plasmid contigs, using curated cutoffs (*–cut_ga*) and other options recommended in the Rfam tutorial: *–rfam –nohmmonly –clanin Rfam.clanin –oskip –fmt 2 -o output.txt –tblout table.txt Rfam.cm input.fasta* (https://rfam.github.io/rfam-tutorials/).

#### Pairwise similarity analysis

Analysis of pairwise similarity between plasmid contigs was carried out using Sourmash v.4.8.8 (Pierce et al. 2019). First, contigs signatures were generated via *sourmash sketch* with k-mer size of 31 and scale value of 1000 (*-p k = 31, scaled = 1000*). Next, the signatures were compared with *sourmash compare* and visualized with *sourmash plot*, using default options for both commands.

#### Semiautomatic annotation of selected plasmid contigs

Selected plasmid contigs were first automatically annotated with Bakta (database version 2024–01–19) (Schwengers et al. 2021). Next, the annotations were manually validated using a combination of blastp from the NCBI BLAST+ suite and HHpred from the MPI Bioinformatics Toolkit webserver (Camacho et al. 2009, Zimmermann et al. 2018).

## Results and discussion

### Selection of sequencing platform has a major impact on assembly outcomes

Samples containing bacterial DNA were isolated from multiple vent fields located in the AMOR in the Norwegian–Greenland Sea (Fredriksen et al. 2019). Sampling sites were characterized by different temperatures, varying from 10°C (sample M4) to 72°C–75°C (samples M5, M11, M19, and M21). Most of the samples were collected from hydrothermal sediments (M4, M10–14, M19–21, and M34) or barite chimneys (M1–M3). Sample M5 was the only one isolated from a white smoker. Full metadata concerning the samples can be found in Table S1.

Following sample collection and processing, DNA isolated from environmental samples was sequenced in two separate batches, resulting in two datasets: MiSeq and NovaSeq. The MiSeq dataset comprised samples M1–M5 and M10–M14 (*n* = 10), which were sequenced using the MiSeq platform in 2 × 300 bp mode. The NovaSeq dataset included samples M19–M21 and M34 (*n* = 4), sequenced using the NovaSeq platform (2 × 150 bp).

**Figure 1.**
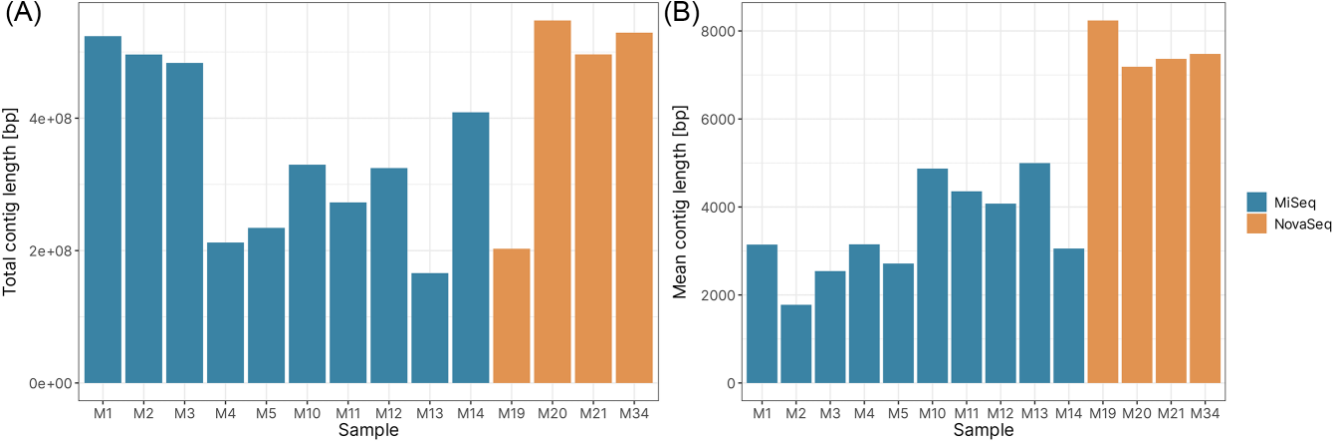
Comparison of assembly results between environmental samples in relation to sequencing platforms. (A) Comparison of the total contig length. (B) Comparison of the mean contig length.

The assembly results revealed notable differences between the two datasets. The NovaSeq samples yielded a higher average total contig length (443 966 701 bp) compared to the MiSeq samples (345 176 277 bp), despite having fewer contigs on average (59 742 versus 116 663) (Fig. 1). This suggests that the NovaSeq platform generated higher-quality data, resulting in longer contiguous sequences. The most striking difference was observed in the average contig length, with the NovaSeq dataset having more than twice the length of the MiSeq dataset (7576 versus 3468). This could be due to the superior quality of NovaSeq data, and/or a coincidentally occurring low number of repeated regions, that usually make it difficult to assemble long sequence contigs from shorter reads (Kusmierek and Nowak 2018).

However, it is important to note that two samples—M13 (MiSeq) and M19 (NovaSeq)—had the smallest total contig lengths. This observation is particularly surprising for sample M19, given that the NovaSeq datasets generally produced higher quality data. This could be attributed to various factors, such as the quality and quantity of the input DNA, the presence of contaminants, or the inherent complexity of the microbial communities in these specific samples. A detailed description of assembly results can be found in Table S2.

Overall, samples obtained from the vent fields at AMOR represent a diverse range of microbial communities adapted to various thermal conditions—from moderate to extreme. The use of two different sequencing platforms, MiSeq and NovaSeq, introduced a significant bias in assembly quality. The NovaSeq dataset seems to be superior, with longer contiguous sequences and higher mean contig lengths. However, the presence of outliers with lower assembly quality in both datasets highlights the need for cautious interpretation of the data.

### Each plasmid identification tools introduces its own taxonomic and functional biases

**Figure 2.**
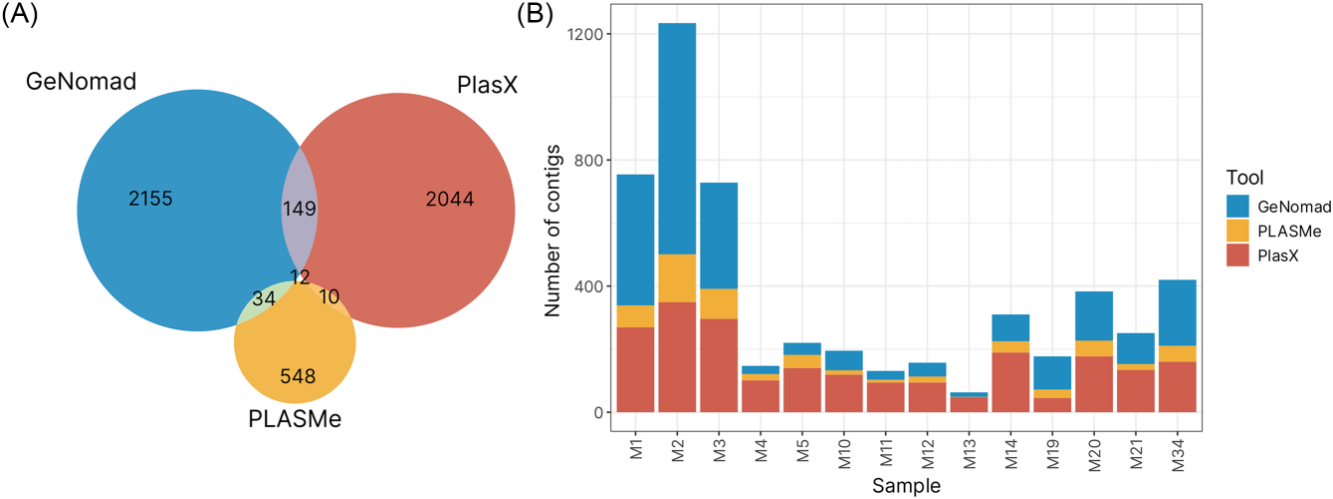
(A) Venn diagram showing the intersection of results of all three plasmid classification tools. (B) Proportion of plasmid contigs identified within each sample by each tool.

The three plasmid identification tools employed in this study—GeNomad, PLASMe, and PlasX—yielded varying numbers of plasmid contigs from 14 environmental samples. GeNomad marked the highest number of contigs assigned as plasmids–2350, followed by PlasX (2215), and PLASMe (604) (Fig. 2A). Overlap between the results of each tool was calculated based on intersection analysis, namely by matching contig names. Remarkably, only 12 contigs were consistently designated as plasmid by all three tools (Fig. 2A). This heavily underlines the importance of tool selection for plasmid prediction, as each tool has its own strengths, weaknesses and biases. GeNomad and PlasX generated results that seem to be more similar, sharing the highest number of contigs among all groups (149). This contrasts with results of PLASMe, which only shared 34 contigs with GeNomad and 10 with PlasX. However, it is important to note that this tool identified a much smaller number of plasmid contigs compared to the other two.

The number of plasmid contigs identified by each tool varied greatly across the 14 environmental samples (Fig. 2B). Overall, samples M1–M3 had the highest number of plasmid contigs identified. On a per-tool basis, GeNomad identified the highest number of plasmid contigs in samples M2 (664) and M1 (397), while PLASMe and PlasX found the most plasmid contigs in samples M2 (151 for PLASMe, 335 for PlasX) and M3 (95, 294 accordingly). Interestingly, all three tools consistently identified the fewest plasmid contigs in sample M13 (GeNomad: 14, PLASMe: 1, and PlasX: 48), with the exception of PlasX, where sample M13 was the second least abundant after sample M19 (45). This consistency may indicate that these samples may have an inherently lower plasmid content compared to other samples, or that the plasmid contigs present in these samples are particularly difficult to identify using the employed tools, either due to their design or the reference data used during the training process.

#### Selection of taxonomic classifiers matters

To determine the taxonomic origin and potential host of the plasmid contigs, two separate classifiers were used and compared to find the optimal result. While correct taxonomic annotation of plasmid sequences is very difficult, given that they often differ in properties such as GC-content and k-mer composition from their host, even a low-level assignment can be greatly beneficial (Aytan-Aktug et al. 2022). For example, determining whether the plasmid originates from bacteria or archaea, can be crucial for its later analysis and annotation.

**Figure 3.**
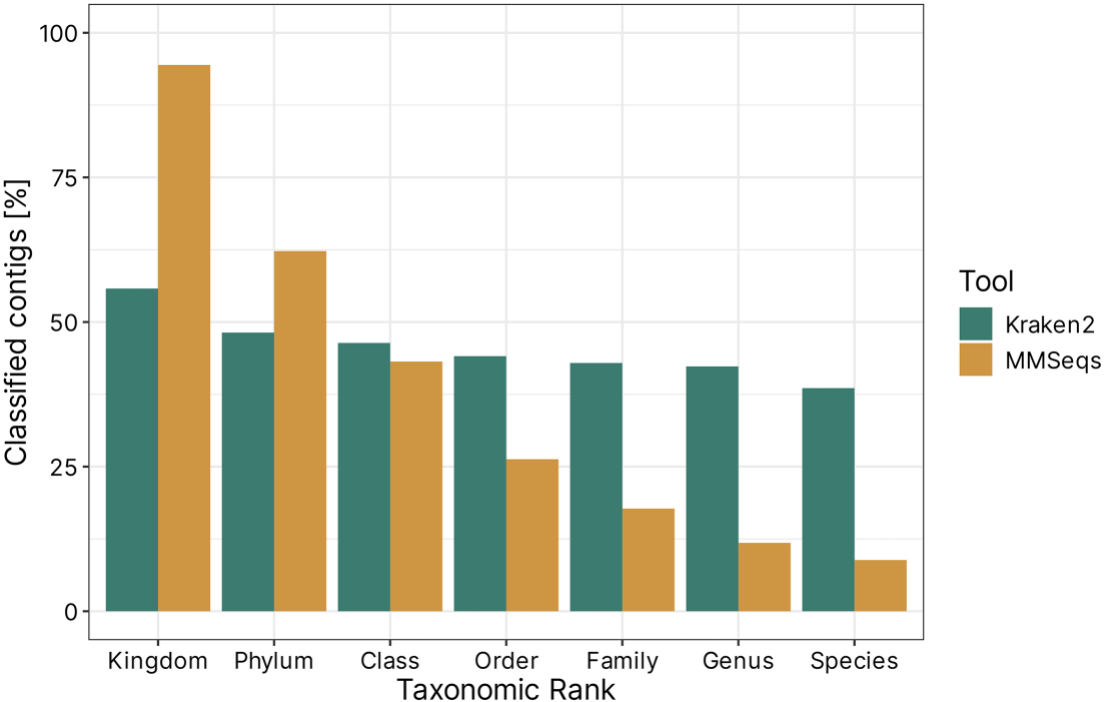
Percentage of all plasmid contigs, which were assigned a taxonomic ID on each level of taxonomy.

Two tools were initially selected for taxonomic analysis: Kraken2 and MMseqs2. To determine which one performs better, all >5000 contigs identified by three plasmid classifiers were assigned taxonomy (Fig. 3). First, we classified the samples using kraken2, using the standard database. Overall, the tool performed well, assigning the lowest taxonomic rank (species) to over 38% of all sequences. Surprisingly, only 55.8% of all sequences were classified at the kingdom level. On the other hand, MMseqs2 with the NR database seemed to fare much better at higher taxonomic levels. The tool assigned a kingdom to 94.4% of all contigs, also outperforming kraken2 at the phylum level (62.3% versus 48.2%). At lower taxonomic levels, the percentage of classified sequences dropped significantly, reaching less than 10% at the species level.

Given the nature of samples analysed in this study, which came from an understudied environment, we believe that the high percentage of low-level annotations provided by Kraken2 may be false. Additionally, since determining the highest ranks, i.e. kingdom and phylum, has the highest impact on downstream analysis, and given that MMseqs2 seemed to outperform Kraken2 on these levels, we decided to use MMseqs as classification tool of choice for further analyses. Furthermore, it must be noted that true plasmid–host association is very hard to determine using solely bioinformatic tools, and usually requires the use of laboratory techniques, such as Hi-C sequencing (e.g. Calderón-Franco et al. 2023).

#### Biggest taxonomic differences between tools are reflected in archaeal diversity

**Figure 4.**
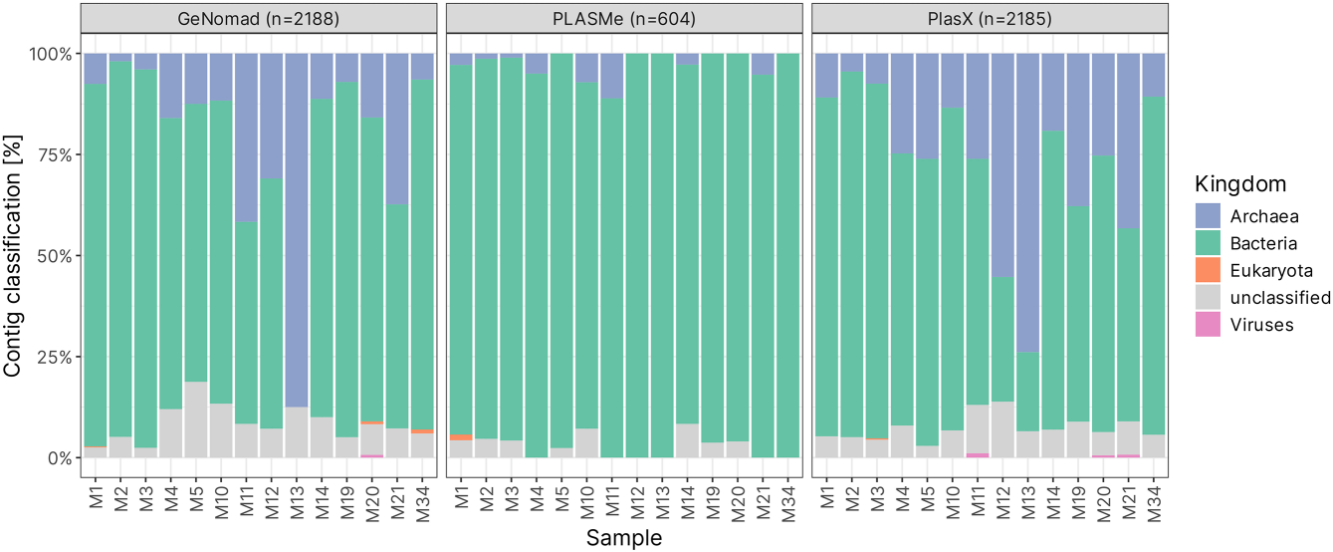
Proportion of plasmid contigs classified into each taxonomic kingdom for each environmental sample.

We analysed the differences in taxonomic composition of plasmid contigs indicated by each plasmid classification tool. In general, contigs originating from bacteria seem to dominate in most environments, regardless of tools used. The exception seems to be sample M13, where both GeNomad and PlasX detected a large proportion of contigs later classified as archaeal (Fig. 4). This aligns well with previous amplicon data, which also showed Archaea as the most abundant in this sample, and with the results from MAG-based metagenomic study (unpublished) (Stokke et al. 2020).

Generally, the results of taxonomic classification of selected contigs seems to be consistent across tools. The data is dominated by bacteria from *Gamma, Delta*, and *Epsilonproteobacteria* classes, even though the proportion of hits vary between tools. Notably, *Flavobacteria* have only been observed in GeNomad output, while PLASMe was the only tool to identify contigs classified as *Bacilli*. Overall, GeNomad detected the most phyla not found in results of other tools (22), followed by PlasX (10) and PLASMe (6). Interestingly, PlasX identified a high proportion of *Anaerolineae*—members of the *Chloroflexi* phylum often found in marine sediments (Blazejak and Schippers 2010) (Fig. S1).

One of the most important differences between the resulting datasets is the minuscule presence of archaeal data in PLASMe output. This may be due to the reference dataset used to train the tool, as it consisted exclusively of bacterial data (Tang et al. 2023).

#### Degree of similarity between proteins from the distinguished plasmid contigs and the reference plasmids varies between tools

To gain a better understanding of which plasmid contig dataset show the greatest degree of similarity to known plasmids, we compared the proteins found on plasmid contigs identified by PlasX, PLASMe, and GeNomad to proteins found on plasmids deposited in the PLSDb database. For each protein found within our datasets, we reported five best hits to the proteins from PLSDb. Next, we analysed the density of hits based on mean coverage between query and subject sequence, as well as % identity.

**Figure 5.**
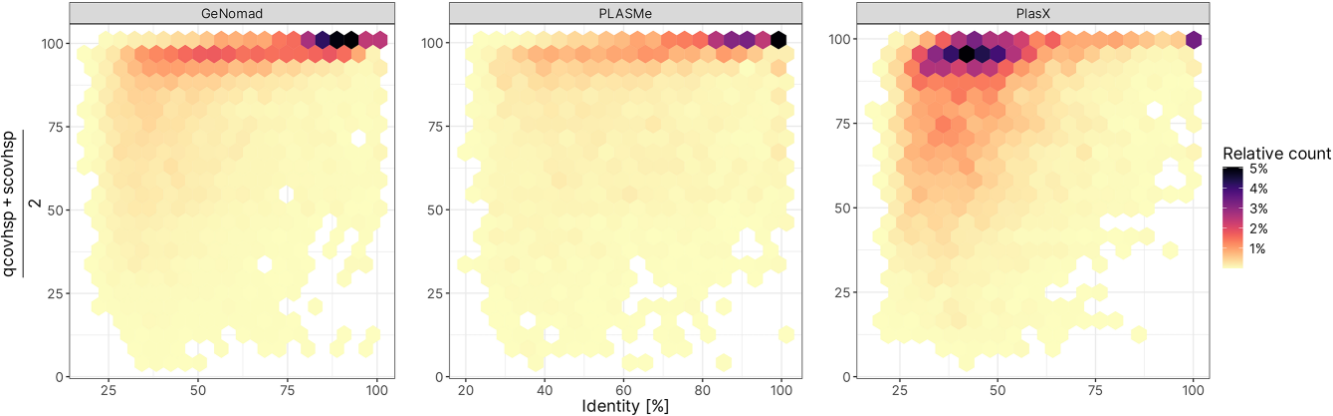
Distribution of identity and mean coverage of hits between proteins identified on plasmid contigs and proteins from the PLSDb database. For each query protein, the top five hits (*diamond -k 5*) were reported.

Our analysis revealed that while the general trends are similar between datasets, there are some notable differences. Overall, a high percentage of all hits showed very high identity and coverage, especially for hits from GeNomad and PLASMe (Fig. 5). Practically no hits were identified near the origin of the density plot, indicating that all sequences showed at least a low similarity to known sequences. Interestingly, for PlasX results, the highest density of hits is located between 30% and 50% of identity, while keeping over 80% coverage. Additionally, a localized maximum can be seen for hits with >99% coverage and identity. This could suggest that this tool was able to detect contigs containing not only the conserved plasmid core, but also the novel genetic load.

Furthermore, while results of search for GeNomad and PLASMe datasets are located mostly within 80%+ coverage range, a significant proportion of hits for proteins originating from PlasX-identified plasmids contigs showed coverage below 75%.

#### Content of plasmid contig datasets varies between tools on functional level

Another very important aspect of biological data, especially for metagenomic datasets, is its functional composition. In order to determine what kind of functional modules and traits can be found within plasmid contigs identified by each classifier, we employed multiple tools, each targeting a different aspect of the metagenome.

**Figure 6.**
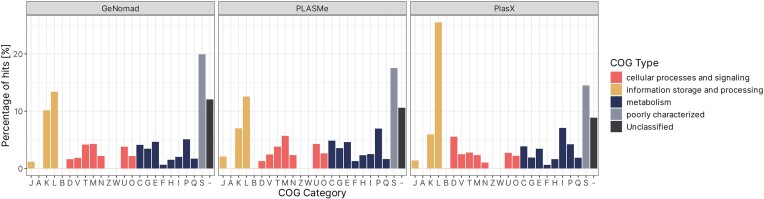
COG types and categories assigned to genes found within contigs classified as plasmid per each classification tool. Annotations for COG categories are as follows: J: translation, ribosomal structure, and biogenesis, A: RNA processing and modification, K: transcription, L: replication, recombination and repair, B: chromatin structure and dynamics, D: cell cycle control, cell division, and chromosome partitioning, Y: nuclear structure, V: defense mechanisms, T: signal transduction mechanisms, M: cell wall/membrane/envelope biogenesis, N: cell motility, Z: cytoskeleton, W: extracellular structures, U: intracellular trafficking, secretion, and vesicular transport, O: post-translational modification, protein turnover, and chaperones, C: energy production and conversion, G: carbohydrate transport and metabolism, E: amino acid transport and metabolism, F: nucleotide transport and metabolism, H: coenzyme transport and metabolism, I: lipid transport and metabolism, P: inorganic ion transport and metabolism, Q: secondary metabolites biosynthesis, transport, and catabolism, R: general function prediction only, and S: function unknown.

First, we used eggNOG-mapper to get an in-depth understanding of the general function of each gene found on plasmid contigs. The first set of information that was of great interest, was annotation with COG categories. A COG category was assigned to 87.0%, 88.6%, and 90.8% of all protein CDS for GeNomad, PLASMe, and PlasX contigs, respectively (Fig. 6). Furthermore, 67.3%, 71.1%, and 76.3% of all CDS were assigned a category other than S (Poorly Characterized), meaning that at least their general function is known. This result suggests that plasmid contigs selected by PlasX show the greatest similarity to known sequences, or that their content is the most similar to data in the COG database. On the other hand, GeNomad had the highest proportion of Unclassified and Poorly Characterized proteins, suggesting a “less conservative” approach. As mentioned before, both results can be beneficial when working with data from extreme environments, as a more conservative approach can guarantee a larger proportion of True positives, whereas an opposite approach can lead to discovery of novel sequences.

On COG category level, genes involved in categories L (DNA replication, recombination, and repair), K (transcription), and P (inorganic ion transport and metabolism; GeNomad and PLASMe) or I (lipid transport and metabolism; PlasX) were most common. Given the type of data—plasmid contigs—this result was desired. In general, proteins involved in DNA metabolism are not only essential for plasmid functioning but are also most conserved and best described. High abundance of genes involved in transcription may be interesting, since they are not so commonly found on plasmids. This could be attributed to presence of prophage regions, genetic load of the plasmids, or contamination with chromosomal sequences. Similarly, presence of proteins responsible for transport and metabolism of either inorganic ions or lipids is expected on plasmid sequences, as they can provide a significant adaptational advantage. On the other hand, proteins from categories A, W, B, and Z were only found in less than five copies for all datasets.

The most striking difference between three analysed datasets was observed for the category D—cell cycle control, cell division, and chromosome partitioning. For GeNomad and PLASMe datasets, proteins from this category constituted about 1.5% of all proteins, whereas for PlasX the percentage was 5.5%. This could be attributed to contamination of the dataset with chromosomal data, but also to presence of proteins responsible for plasmid partitioning and maintenance. Further analysis of the D category revealed that most proteins were classified as tyrosine recombinases (Fig. S2). Interestingly, while 133 of those proteins were found in the PlasX dataset, only five were identified in GeNomad data, and none were present in PLASMe plasmid contigs.

Additionally, 54 proteins marked as “involved in chromosome partitioning” were identified in PlasX data. The difference was not as great as for recombinases, with GeNomad also outputting plasmid contigs containing 22 proteins with the same classification. Again, PLASMe did not contain any such proteins. Overall, this analysis revealed that PlasX showed a significant bias towards certain proteins from the COG D category, especially *xerC* and *xerD* site-specific tyrosine recombinases, and proteins involved in chromosome partitioning. GeNomad results were more moderate and did not show any significant trends in terms of COG category D proteins. PLASMe dataset contained the least proteins from this category (12), compared to other tools (144 for GeNomad and 406 PlasX), but relative count did not differ significantly from GeNomad (1.28% versus 1.61%), and can be attributed to overall smaller number of plasmid contigs.

Yet again, because of the nature of the dataset analysed in this study it is hard to determine whether the biases described above are a result of contamination or novelty. Presence of site-specific tyrosine recombinases is usually correlated with integration of prophages into sequences, which can happen in any part of the genome. This hypothesis could be supported by the results of taxonomic analysis, as PlasX was the tool with the highest number of contigs classified as viral. Similarly, proteins involved in chromosome partitioning can be responsible for chromosome partitioning, or may be mis-annotated, and in fact be involved in plasmid partitioning systems. This is quite likely, given the similarity between both types of proteins. Interestingly, PLASMe output does not contain most of the proteins described above. This could be a sign of high precision of the tool, as proteins that are likely found on chromosomes are not found in this dataset. On the other hand, typical plasmid proteins, like *parA*, toxin–antitoxin systems, and plasmid maintenance proteins are also absent from this dataset, which could indicate lower recall of this tool.

**Figure 7.**
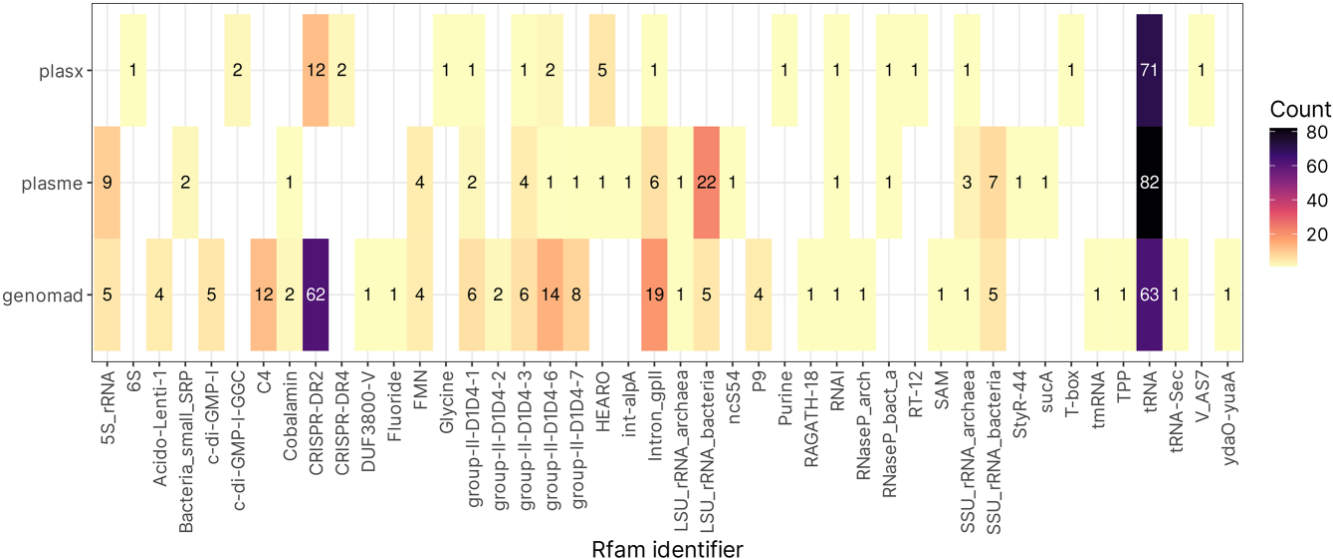
Heatmap showing the count of RNAs identified within data originating from each plasmid identification tool. Identification was performed using Rfam database and Infernal.

Next, we performed identification of RNAs found on plasmid contigs identified by all three classifiers. The classification was carried out using the Rfam database with Infernal, using cutoff values set by Rfam curators when creating families. Initial analysis revealed a wide array of ncRNAs found in all three datasets. For all tools, tRNAs were the most abundant type of RNA. Additionally, RNAI, an element typically found on *ColE1*-like plasmids, was also identified in all three datasets, although only in one copy (Helmer-Citterich et al. 1988). Interestingly, a multitude of group II catalytic introns was also found in each tools’ output—including general (Intron_gpII) and specific hits (group-II-D1D4-1,3,6). Finally, archaeal small subunit ribosomal RNA was also found in all datasets (Fig. 7).

Overall, there were no significant differences in results of search between datasets, except for CRISPR-DR elements, found in large abundance in GeNomad data, and to some extent in PlasX, but not in PLASMe. Interestingly, both archaeal and bacterial large subunit ribosomal RNA was identified in PLASMe and GeNomad data, but not PlasX.

For GeNomad, CRISPR RNA direct repeat element 2 was almost as abundant as tRNA (62 hits and 63 hits, respectively), suggesting that the tool may be biased towards contigs containing this element. CRISPR direct repeats were the third most abundant element for PlasX, with 14 hits. Further analysis revealed that the repeats are located on six contigs, two carrying only one copy, one with two copies, and contigs with 11, 27, and 34 copies. Further analysis revealed no Cas genes located on these contigs. However, *Cas* and CRISPR-related proteins were identified on other plasmid contigs originating from the same environment, indicating that working systems could be present *in vivo* (for example, sample M11, containing a contig with 34 DRs, also contained *cas1, cas2*, and *cas6* proteins, as well as other CRISPR-related proteins). Having considered this information, as well as the fact that the plasmid contigs come from metagenomic assemblies and may not be complete, it is nearly impossible to determine if the systems are in fact functional or not, especially without applying laboratory experiments.

Finally, we decided to identify typical plasmid proteins, involved in MOB, MPF, and REP. For this, we utilized MOB-suite databases (Robertson and Nash 2018). Initial analysis revealed a low number of proteins belonging to any of the groups (Fig. S3).

First, we scrutinized the MOB proteins. We were able to identify proteins from five different MOB groups (F, H, P, Q, and V), but only one of them (P) was identified in all three datasets. Overall, the most MOB proteins were identified in GeNomad dataset (18), while PLASMe had the same number as PlasX (10), despite a much lower count of contigs in output dataset. The only MOB type identified in all datasets was MOBP.

Next, the protein group which showed the most striking differences—MPF proteins. This group was the most numerous. Again, GeNomad had the most hits (69), followed by PlasX (44) and PLASMe (6). Only three types of MPF proteins were found—F, T, and unknown—with MPF-F and MPF-T being found in all three datasets, albeit in much lower counts within the PLASMe output.

Results of REP analysis were scattered, with no protein cluster gathering more than four hits from one tool. Overall, the results of this analysis are quite surprising. Given the fact that a total of over 5000 plasmid contigs were analysed, obtaining 225 hallmark plasmid genes is appalling. Once again, this result can be attributed to many factors. Most likely includes database bias, lack of reference data, and incompleteness of contigs (i.e. a full plasmid sequence can be split into multiple parts, out of which only one will contain a REP system). Nevertheless, further analysis would have to be conducted in order to determine what partitioning and mobilization systems can be found in the microbial communities of the AMOR ridge.

### Characterization of majority voting plasmid contigs

In order to address the discrepancies between the results of all three tools and remove their respective biases, a majority voting system was used to determine contigs that have a maximally high probability of actually originating from plasmid DNA. Contigs classified as plasmid by at least two out of three tools were selected, resulting in a dataset containing 205 plasmids, with 12 of them being selected by all three tools. These high-confidence plasmids present a valuable resource for an in-depth analysis of the taxonomic, functional, and metabolic diversity within the AMOR microbial communities. Surprisingly, 41 contigs carrying hallmark plasmid genes (MOB, REP, or MPF) identified with MOB-scan were not included in this set. However, most of the hits were of poor quality, and as such, those contigs were not further considered. A series of analyses analogous to that performed beforehand using datasets from three plasmid classification tools was performed. First, we looked into the taxonomic classification and diversity between environments. Next, we scrutinized the functional and adaptational value of the plasmid contigs in question, highlighting their role in adaptation to extreme conditions. Finally, we tried to fund similarities to plasmid sequences available in public databases.

#### High-confidence plasmid contigs vary in size and GC content

**Figure 8.**
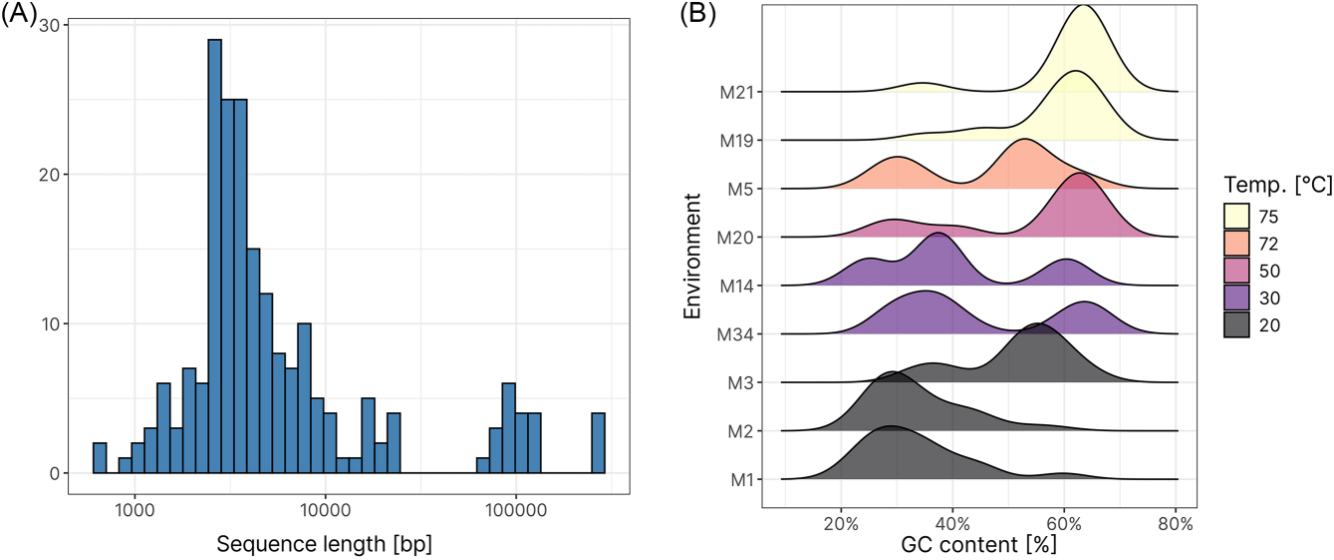
(A) Histogram of sequence length. (B) Ridge plots displaying GC content by environment in plasmids from majority voting dataset. For GC content plots, environments M4, M10, and M12 were excluded, as they contained two or less plasmid contigs.

Typically, plasmids range in size from around 700 bp to 400 kb, with some (e.g. mega-sized plasmids in *Alphaproteobacteria*) reaching over 1 Mb in size (Thomas and Summers 2008, Dziewit et al. 2014, Ciok et al. 2016, Frage et al. 2016). Plasmid contigs identified via majority voting varied in size from 611 bp to 251 kb, staying within reasonable range for predicted plasmids. Average length was 18.1 kb, while median length was 3740 bp (Fig. 8A). While this could potentially be a result of mostly small plasmids being present in the environment, it is more likely a result of fragmentation of sequences. Mean GC content of all 205 plasmid contigs was 58.37%, but a more detailed analysis revealed that most plasmids have a GC content of either around 30% or 60%. Since GC content of sequence is usually linked with temperature of the environment (higher temperatures mean higher GC content), we also analysed how the GC content changes based on the temperature of the sampling site (Hu et al. 2022) (Fig. 8B). For environments with the highest temperature (75°C), a peak was observed at ∼65% GC content. As the temperature dropped, the height of this peak started dropping, while a second peak, located at ∼30% GC content started appearing, increasing in size, reaching its maximum at 20°C. This clearly showcases how temperature affects the GC content of plasmid contigs.

#### Taxonomy of plasmids from DSHVs is biased toward *Pseudomonadota* and *Campylobacterota*

One of the most important characteristics of plasmid sequences is their taxonomy, or association with the host. However, it's crucial to remember that plasmids are mobile and may be exchanged between hosts. To determine the taxonomy of plasmid contigs selected via majority voting, we applied the same methodology as before, using MMseqs2 easy-taxonomy module with NR database as reference. Initial analysis revealed that over 96% of all plasmid contigs were classified within any taxonomic rank. The percentage dropped rapidly with decreasing rank, with 75% plasmid contigs classified at phylum level, 58% at class level, down to 9% at species level (Fig. S4A). In general, the dataset is dominated heavily by *Bacteria*, with only one out of 205 sequences classified as Archaeal. The annotation of this sequence only reached the phylum rank—*Euryarchaeota*. Furthermore, seven sequences were not assigned to any Kingdom, and 193 plasmid contigs were classified as Bacterial. Classification of all bacterial plasmid contigs on phylum level can be seen on panel B of Fig. S4.

The most abundant phyla were *Pseudomonadota* and *Campylobacterota*. On family level, *Burkholderiaceae* were observed the most frequently, with 29 plasmid contigs assigned to this rank. The next most abundant family was *Arcobacteraceae*, with five observations. This family is unique within the *Campylobacterota* phylum, as it is found in an unusually wide range of environments, including vents at AMOR (Fera et al. 2004, Urich et al. 2014, Dahle et al. 2015, Stokke et al. 2015, Steen et al. 2016). One of the species within this family, *Arcobacter sulfidicus*, produces filamentous sulfur, which may indicate its pivotal role in formation of white sulfur mats, useful in anchoring microbes to rocky surfaces affected by flow of hydrothermal fluids (Wirsen et al. 2002). Other families known to metabolize sulfur, namely *Sulfurimonadaceae* and *Chromatiaceae* were also identified (Hubas et al. 2013, Han and Perner 2015). Other than that, plasmid contigs were classified as originating from families *Methylococcaeae* (two contigs), *Enterobacteriaceae* (one), *Paracoccaceae* (one), *Roseobacteraceae* (one), and *Wenzhouxiangellaceae* (one).

Overall, we believe that the results presented are in line with the fundamental role of sulfur species in forming and nourishing the bacterial communities found in deep-sea environments (Urich et al. 2014, Dahle et al. 2015, Stokke et al. 2015, Steen et al. 2016). Another fact that must be considered is the relatively low percentage of successfully classified sequences, especially at lower taxonomic levels. This stems from the under-representation of data from extreme environments in public databases, as well as lack of experimental data confirming their taxonomy.

#### Plasmids may provide a plethora of functional advantages to their hosts

**Figure 9.**
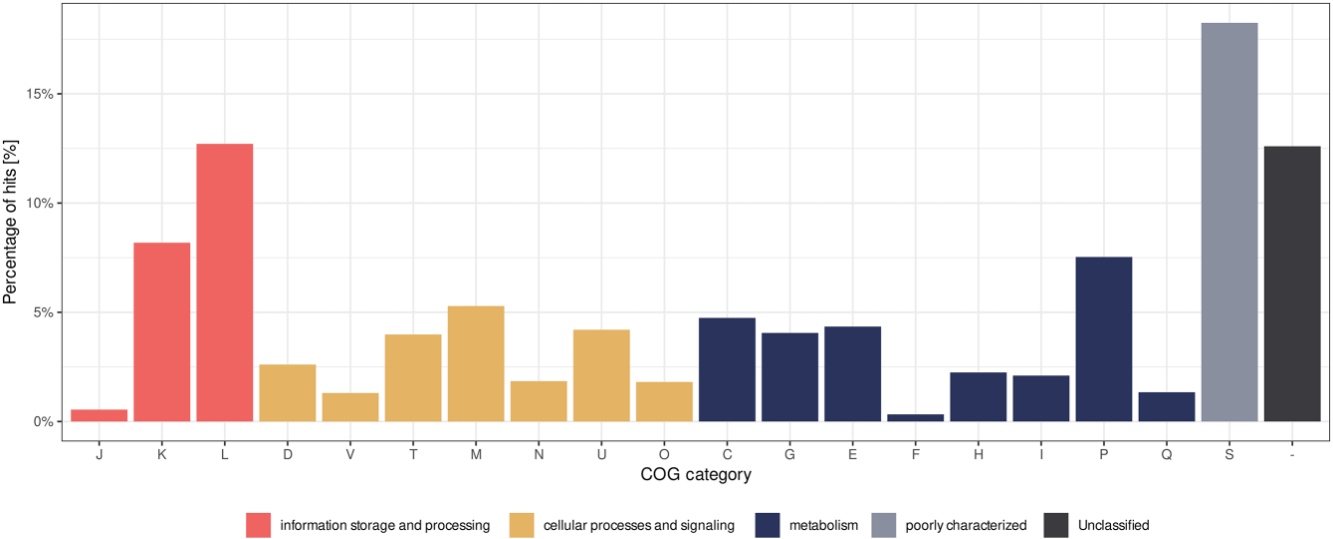
COG categories assigned to genes found within contigs classified as plasmid by at least two classification tools. Annotations for COG categories are as follows: J: translation, ribosomal structure and biogenesis, K: transcription, L: replication, recombination, and repair, D: cell cycle control, cell division, and chromosome partitioning, V: defense mechanisms, T: signal transduction mechanisms, M: cell wall/membrane/envelope biogenesis, N: cell motility, U: intracellular trafficking, secretion, and vesicular transport, O: post-translational modification, protein turnover, chaperones, C: energy production and conversion, G: carbohydrate transport and metabolism, E: amino acid transport and metabolism, F: nucleotide transport and metabolism, H: coenzyme transport and metabolism, I: lipid transport and metabolism, P: inorganic ion transport and metabolism, Q: secondary metabolites biosynthesis, transport, and catabolism, and S: function unknown.

To gain an insight into the metabolic functions of plasmid contigs, we analysed the presence and relative abundance of proteins within each COG category, as well as looked into specific categories to reveal what evolutionary advantages they may provide. Similar to results coming from each plasmid classifier, plasmid contigs selected via majority voting system were enriched with genes from COG categories L, K, and P (replication, recombination, and repair; transcription; inorganic ion transport and metabolism, respectively), suggesting that, apart from functions related to plasmid maintenance and transmission, functionality related to utilization of novel, inorganic energy sources is the most enriched within plasmid contigs (Fig. 9).

In order to determine the exact functionality carried by plasmid-encoded proteins, we next analysed the output of eggNOG-mapper in terms of both Pfam hits and KEGG hits. For both categories, if a protein was annotated with more than one ID, the IDs were split and treated as separate entries. 2575 proteins, originating from 205 plasmid contigs, were assigned 3875 nonoverlapping Pfams and 2728 KEGG KO terms.

Overall, most abundant protein families (i.e. Pfams) identified in the majority voting plasmid dataset were related to phage biology and transmembrane transport. Specifically, we identified 73 hits to phage integrases (PF00589) and 29 hits to their N-terminal SAM-like domain (PF02899), as well as 32 resolvases (PF00239), often encoded within Tn*3*-like transposons (Heffron et al. 1979). In terms of transmembrane transport and substrate binding, we identified a wide range of domains, with the LysR substrate binding domain (PF03466) having the most hits (47). This domain can have a critical role in ensnearing substrates available in the environment, including amino acids, sugar phosphates, organic acids, metal cations and many more (Matilla et al. 2022). Potential role of plasmids in acquisition of substrates from the environment can be further confirmed by the presence of numerous copies of domains involved in transport of various substrates, including ABC transporters (PF00005, 43 hits), major facilitator superfamily (MFS) members (PF07690, 40 hits; PF05977, 4 hits; PF06779, and 4 hits), ACR family membrane proteins (PF00873, 32 hits), and binding-protein-dependent transport system members (PF00528, 30 hits). Additionally, many regulatory domains were found as well, such as the regulatory helix-turn-helix protein from LysR family, matching the LysR substrate-binding domain (55 hits, PF00126); 34 general response regulator domains (PF00072), and more. We also identified domains responsible for binding and transport of metals (such as CopB and copper oxidases; ChrB, and other chromate transporters), multiple 2Fe–2S iron–sulfur cluster binding domains (fer2, fer4_4,12,14), conjugal transfer proteins (*traCEFGHLN*), DNA methylases and polymerases, and multiple DDE transposases. Overall, we were able to identify 589 unique domains, and 421 proteins were not assigned any Pfam domain.

Despite basing on the same input dataset, the results of KEGG KO annotations present a different functional landscape compared to Pfam. Here, by far the most numerous annotation belongs to partitioning proteins (K03496–chromosome partitioning protein–42 hits; and K03497–ParB chromosome partitioning protein–24 hits) and chemo- and aerotaxis proteins (K03406–methyl-accepting chemotaxis protein–20 hits; K03776–aerotaxis receptor–16 hits; K05874–methyl-accepting chemotaxis protein I, serine sensor receptor–17 hits; and K05875–methyl-accepting chemotaxis protein II, aspartate sensor receptor–12 hits). On the other hand, some similarities were retained, such as presence of copper transport and resistance proteins (K17686, K07156, and K07233), MFS proteins (K08191, K03535, K08225, and K08369), and conjugal transfer proteins (K12056–K12072). Here, the number of proteins with no database ID assigned was much higher compared to Pfam, as 1429 proteins were not matched with any KEGG KO, and the rest were assigned 310 unique KO numbers.

#### Choice of sequencing platform significantly affects outcomes of functional annotation

While differences observed between different sources of annotation (Pfam versus KEGG) are not drastically big, they are much more noticeable when comparing annotations between samples originating from different sequencing platforms. Namely, the number of unique Pfam and KEGG annotations assigned to proteins originating from each environment, is much higher for NovaSeq samples compared to MiSeq data (Fig. S5). This is especially significant for samples M2 and M3, which, despite containing a high number of plasmid contigs (62 for M2, highest of all samples, and 27 for M3, third highest), have a much smaller proportion of unique database IDs within them. Samples M4, M10, M12, and M14 only contain a minimal number of plasmid contigs (2, 2, 1, and 4, respectively).

While differences in environmental diversity could contribute to variations in annotation, the results from section "Selection of sequencing platform has a major impact on assembly outcomes" suggest that sequencing methodology plays a significant role. The higher quality and quantity of data produced by NovaSeq may allow for more comprehensive assembly and annotation of plasmid sequences, revealing a greater diversity of functional genes. Conversely, the shorter, potentially fragmented assemblies obtained from MiSeq data may limit the detection and characterization of certain plasmid-borne genes, leading to an underestimation of functional diversity. However, the observed variability within MiSeq samples suggests that the inherent plasmid content and community structure within these environments could also contribute to the observed differences.

In conclusion, the functional analysis of high-confidence plasmid contigs from DSHVs revealed a diverse array of genes involved in various metabolic processes. While core plasmid functions like replication, transcription, and inorganic ion transport were enriched, the presence of numerous genes related to phage biology, including integrases and resolvases, suggests complex interactions between plasmids and phages in this ecosystem, possibly interplay between these elements leading to formation of plasmid-like prophages or integration of phages within plasmids or exchange of genetic modules. This interplay may be crucial for plasmid maintenance, horizontal gene transfer, and the acquisition of novel adaptive traits. The abundance of genes related to transmembrane transport and substrate binding further indicates a pivotal role of plasmids in facilitating nutrient uptake and adaptation to the unique geochemical conditions of hydrothermal vents. Notably, a significant proportion of proteins lacked annotations in both the Pfam and KEGG databases, highlighting the understudied nature of this environment and the potential for novel gene functions yet to be discovered. It is also important to acknowledge that the observed functional profiles may be influenced by the biases of the individual plasmid classifiers, each potentially favoring certain types of plasmids based on their training data and algorithms.

### In-depth analysis of 12 plasmid contigs selected by all tools

**Figure 10.**
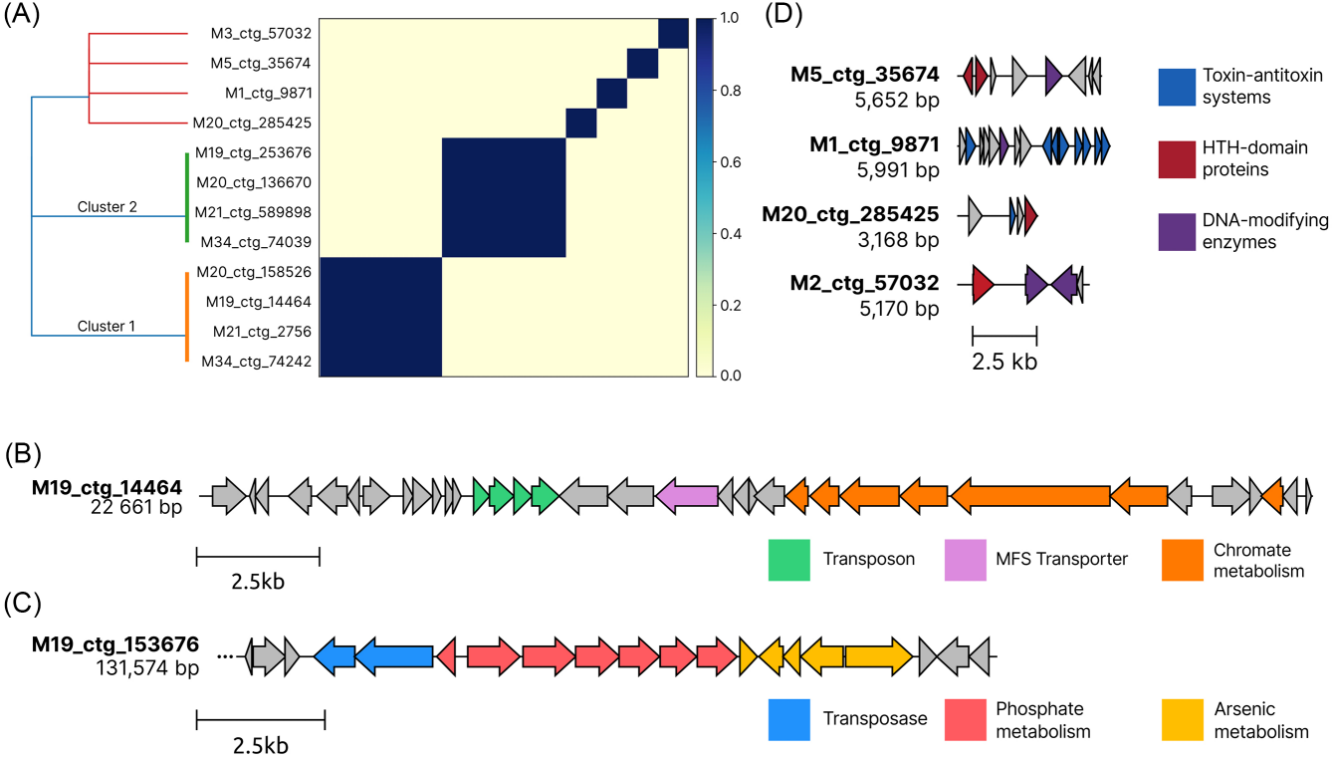
(A) Heatmap showing pairwise similarity between 12 plasmid contigs, basing on estimated Jaccard similarity. (B) Linear map of the plasmid contig M19_ctg_14 464, with features classified into general categories. Arrows indicate predicted genes. For genes with multiple potential classifications, the most specific was selected. (C) Schematic representation of the putative genomic island identified on plasmid contigs from the cluster 2. (D) Linear representation of plasmid contigs M5_ctg_35 674, M1_ctg_9871, M20_ctg_28 5425, and M2_ctg_57 032. The DNA-modifying enzymes category includes integrases, recombinases and DNA polymerase.

The last step in analysis of plasmid data obtained from tested metagenomic samples was to take an indepth look at 12 plasmid contigs selected by all three tools used in the study. All of the sequences can be found in supplementary file F1. Plasmid contigs contained within this dataset should have a maximally high likelihood of being actual plasmids/fragments of plasmids. The first step in this analysis was to compare the plasmid contigs to determine their similarity, and potentially find clusters of similar plasmids. In order to do so, we used k-mer-based tool sourmash (Pierce et al. 2019). Results showed that the 12 plasmids formed two clusters of size four, containing nearly identical sequences (estimated Jaccard similarity index equal to 1). For the remaining four contigs, no similarity to any other plasmid contig from this dataset was found (Fig. 10A). Notably, plasmid contigs which formed clusters 1 and 2, originated from environments M19, M20, M21, and M34, sequenced using the NovaSeq platform; and only three plasmids in this dataset originated from MiSeq data. Once again, the importance of sequencing platform selection and its effect on results of downstream analyses is provided.

#### Plasmid contigs from cluster 1 provide chromate and superoxide resistance

The cluster 1 contained contigs M19_ctg_14 464, M20_ctg_158 526, M21_ctg_2756, and M34_ctg_74 242, each 22 261 bp long, and with GC content of 64.86%. Each contig contained 29 protein CDS. Basing on the results of MMseqs2 taxonomic analysis, the contigs were classified into Pseudomonadota phylum. A blastn search confirmed very high similarity (>99% identity and coverage) to plasmids originating from other members of this *phylum*, namely *Ralstonia* strains, although the target sequences were much longer (190–340 kb). Linear map of the plasmid contig M19_ctg_14 464 as a representative of all four contigs found within this cluster can be seen in Fig. 10(B).

The next step was to perform functional annotation of CDS, which was carried out using HHpred. First, we tried to identify hallmark plasmid genes. Unfortunately, no REP, MOB, or MPF genes were identified within the sequences. Similarly, GeNomad, which uses a custom database of hallmark plasmid genes, also identified no such features. Despite that, the contig did contain two potentially interesting modules. First, a transposon-like module, containing 4 genes (coordinates 5585–7327 bp), containing an integron gene cassette protein, a transposase, a prophage protein and site-specific DNA recombinase SpoIVCA/DNA invertase PinE. The functionality and exact role of this module is hard to determine, as the annotations for proteins in this element were of low quality. Second, a chromate-resistance module was identified. This module encodes six proteins, including a periplasmic adaptor subunit of RND efflux transporter, CzcA family RND efflux transporter, two chromate resistance proteins, and a superoxide dismutase. Together, these proteins have a potential to form a fully functional chromate-resistance mechanism, which includes reduction, binding and transport of chromate. Interestingly, a similar genetic module was reported by Branco et al. (2008). In their work, the authors describe an operon of analogous structure and confirm its function in chromate and superoxide resistance. Furthermore, the element described in their work was located within the *TnOtChr* transposable element, similar to how the chromate resistance genes identified in this work are located near a transposase gene. Another two genes, potentially involved in metal resistance are located both upstream and downstream—a periplasmic heavy metal sensor, and a MFS transporter. A putative nucleotidyltransferase, followed by a putative toxin of the MNT–HEPN system was also found. Upon further analysis the nucleotidyltransferase protein showed similarity to *mtnA* adenylyltransferase, further suggesting the completeness of this toxin–antitoxin system (Yao et al. 2020).

#### Cluster 2 plasmid contigs contains a genomic island related to phosphate transport and arsenate metabolism

The second identified cluster, marked on Fig. 10(A) with color green, groups much larger contigs. Contigs M19_ctg_253 676, M21_ctg_589 898, and M34_ctg_74 039 are 131.5-kb long, while contig M20_ctg_136 670 is about 1 kb longer, i.e. 132.6 kb. The additional fragment, not found in other contigs, contains two predicted genes. GC content was identical for all four contigs at 59.9%. Taxonomic classification of these contigs placed them in the *Burkholderiaceae* family. Unlike with cluster 1, a blastn analysis showed no significant similarity between the plasmid contigs from this cluster and sequences from the NT database.

Akin to cluster 1, the first goal of analysis of this set of plasmid contigs was to identify hallmark plasmid genes. This time, the identification was successful. Most notably, genes responsible for plasmid partitioning (*parAB*) and conjugation (*virB-*like) were found, cementing the origin of those sequences as plasmid. A full list of 10 genes marked as hallmark by GeNomad can be found in Table S3. Furthermore, manual analysis of nearby loci revealed the presence of more MPF-related genes, hinting that the system could be complete. A highly interesting feature of distinguished plasmid contigs is a putative genomic island, located near the end of the contig (Fig. 10C). Downstream, the island is delimited by two IS*21*-like elements encoding a transposase and a helper ATPase. Following these, a *pstSCAB* and *phoUB* genes are present, constituting a high affinity and velocity phosphate transport system along with its regulator (Shinagawa et al. 1983, Yuan et al. 2006). Additionally, the genomic island, as observed based on localized drop in GC content, contains a series of *ars* genes (*arsR, arsI, arsR*, and *arsH*), followed by a MFS transporter, forming a structure similar to that described by Muller et al. (2007), although the presence of two *arsR*-like regulatory proteins is uncommon. Those genes could provide a critical advantage to the host of this plasmid, by enabling As respiration. Zhang et al. (2023) proved that they may play a pivotal role in metabolism of microorganisms found in deep sea cold seep sediments, as well as global carbon and nitrogen cycling. Compared to mechanisms described in this study, the module is missing genes responsible for As(V) reduction and oxidation methylation of As(III).

Other than that, various other enzymatic proteins were identified, including, but not limited to aldehyde dehydrogenase, aspartate carbamoyltransferase, and chemotaxis protein, putatively providing various metabolic advantages to host cell (Schalk et al. 2009, Lipscomb and Kantrowitz 2012, Shortall et al. 2021). Notably, the quality of annotation of this plasmid contig was relatively low, with 46 out of 144 genes found within the plasmid marked as hypothetical proteins.

#### Small plasmid contigs encode many toxin–antitoxin systems suggesting their selfish nature

Other than the eight plasmid contigs, forming clusters 1 and 2, which were described above, four unique, nonclustering plasmid contigs were identified as well. Representation of all four contigs can be seen on panel D of Fig. 10.

First, contig M5_ctg_35 674 with 50.48% GC content, was described. Taxonomic classification indicated the LCA of this sequence as order *Enterobacteriales*. The length of this sequence equals 5.6 kb, and only eight predicted genes were identified within it. Among these, three proteins were marked as hypothetical. We also identified a site-specific DNA recombinase SpoIVCA/DNA invertase PinE, inovirus-type Gp2 protein and two helix-turn-helix domains containing proteins. However, the most notable is the presence of heat shock protein C (HSP C) and RNAI ncRNA sequences. The latter indicates the plasmid is a ColE1-like replicon, while the presence of a HSP protein could provide a significant environmental advantage, given that sample M5 was collected from an environment with temperatures reaching 72°C.

Next, contig M1_ctg_9871—a 5.9-kb sequence with 15 predicted genes and 40.73% GC content, was distinguished. The low GC content, especially compared to other plasmids, can be somehow explained, as sample M1 was collected from an environment with the lowest temperature (20°C). The contig was only classified as bacterial. Among the identified 15 genes, several toxin–antitoxin related proteins were found, including two complete toxin–antitoxin systems (*cddAB* and *hicAB*) and two TA-related proteins (*higA* family addiction module antidote protein and type II toxin–antitoxin system PemK/MazF family). Additionally, a pair of *vapC* ribonuclease, which is a toxin in the *vapBC* system, and putative (anti)toxin protein, located in the upstream ORF, was identified. The putative protein showed moderate similarity to multiple toxin and antitoxin proteins from type II systems, and it could form another TA system within this plasmid. There is also a polymerase beta-like protein and a nucleotidyltransferase, both of which can be involved in base excision repair of DNA (Krokan and Bjørås 2013). Presence of such a system could greatly benefit the host, provided that DNA damage is frequently occurring in the environment.

Plasmid contig M20_ctg_285 424 is the smallest of 12 selected contigs within this dataset. It also has the lowest GC content of just 27.65%, while it originates from a moderately hot environment (50°C). The contig is 3.1 kb long and only encodes four protein-CDS, two of which are hypothetical proteins. The other two are a replication protein and *vbhA* antitoxin of the *vbhAT* toxin–antitoxin system.

Finally, plasmid contig M3_ctg_57 032, is another small sequence (5.1 kb) containing only four open reading frames. Among those, two are integrases, one protein is a HTH-domain containing protein with unknown function, and the final one is a hypothetical protein. Based on the genetic content of this sequence, it is possible that it is not a plasmid, despite being selected by all three tools. Possibly this is a fragment of a larger replicon.

## Conclusions

In this study, we explored the diversity and ecological roles of plasmids in DSHVs located at AMOR in the Norwegian–Greenland Sea—a unique and understudied environment. We first analysed the impact of the choice of sequencing platform and concluded that it significantly impacted the assembly and subsequent functional annotation of plasmid contigs, with NovaSeq data providing greater resolution and uncovering a wider range of functional diversity compared to HiSeq data.

Next, our comparative analysis of three state-of-the-art plasmid identification tools (PlasX, GeNomad, and PLASMe) revealed significant differences in taxonomic composition, degree of similarity to known plasmids and functional content between datasets originating from each classifier. Each tool exhibited different strengths and biases, likely derived from diverse methodologies and reference datasets used during their development.

GeNomad identified the highest number of plasmid contigs and showed a “less conservative” approach, as evidenced by the higher proportion of poorly characterized and unclassified proteins. This suggests that GeNomad could perform better when working with novel sequences, for example originating from extreme environments. However, it also demonstrated a potential bias toward contigs containing CRISPR elements, which warrants further investigation.

PlasX exhibited the greatest similarity to known sequences and COG database entries, suggesting a more conservative approach, yet it identified a number of sequences similar to GeNomad. The tool identified a high proportion of proteins involved in cell cycle control, cell division, and chromosome partitioning, particularly site-specific tyrosine recombinases and proteins involved in chromosome partitioning. While this could be attributed to contamination with chromosomal data, it may also indicate the presence of prophage regions or plasmid partitioning systems.

PLASMe identified a significantly lower number of plasmid contigs compared to GeNomad and PlasX, displaying high precision by excluding proteins typically found on chromosomes. However, the absence of characteristic plasmid genes, such as parA, toxin–antitoxin systems, and plasmid maintenance systems, indicates a lower recall. Additionally, PLASMe’s output was notably lacking in archaeal diversity, most likely due to the exclusively bacterial reference dataset used in its training. To mitigate the aforementioned biases, we created a high-confidence plasmid dataset, obtained through a majority voting approach, which unveiled a diverse array of genes involved in core plasmid functions, phage interactions, nutrient acquisition, and stress response. This functional landscape reflects the complex interplay between plasmids and their microbial hosts in the extreme conditions of deep-sea vents, suggesting an essential role for plasmids in facilitating adaptation and survival. The presence of numerous poorly annotated or novel genes underscores the vast untapped genetic potential of this unique ecosystem. Notably, many plasmid contigs were equipped with toxin–antitoxin systems, responsible for dependence of cell survival on a mobile genetic element, which highlights the role of “selfish DNA” within the studied extreme environments.

Overall, our findings contribute to a deeper understanding of plasmid ecology in DSHVs, revealing the importance of considering both biological and methodological factors when investigating these complex microbial communities. The observed functional diversity highlights the potential for plasmids to drive adaptation and evolution in extreme environments, offering valuable insights into the ecological roles of these mobile genetic elements. Further research, including experimental validation of predicted gene functions, will be crucial for unraveling the full extent of plasmid-mediated processes in these ecosystems. Additionally, the development of refined plasmid identification tools specifically tailored to challenging metagenomic datasets from extreme environments will enhance our ability to comprehensively characterize the (meta)plasmidome and uncover its hidden features.

## Supplementary Material

fiae124_Supplemental_File

## Data Availability

The sequence data used in this study has been submitted to NCBI BioProject (http://www.ncbi.nlm.nih.gov/bioproject) under BioProject accessions: PRJNA587885, PRJNA785779, PRJNA785780, PRJNA785781, PRJNA785783, and PRJNA801110.
