# Supplementary material for: Landscape of the metaplasmidome of deep-sea hydrothermal vents located at Arctic Mid-Ocean Ridges in the Norwegian–Greenland Sea: ecological insights from comparative analysis of plasmid identification tools
Source: FEMS Microbiol Ecol. 2024 Sep 13;100(10):fiae124. doi: 10.1093/femsec/fiae124 (PMC11451466; doi:10.1093/femsec/fiae124)
Supplement: fiae124_Supplemental_File [file fiae124_supplemental_file.pdf]

Table S1. Metadata regarding the samples collected from vent fields located in the Arctid Mid-Ocean Ridge

Short name	Sample ID	Sample type	Temperature	Substrate	Geographic location*	Description	Sequencing technology (year)	Bioproject	BioSample	Reference
M1	10ROV5WB	Barite chimney	20	none	LCVF	Middle section; white barite; diffuse flow	Illumina MiSeq 300bp paired-end (2016)	PRJNA587885	SAMN13352060	Steen *et al.*, 2016
M2	15AGR09BS	Barite chimney	20	none	LCVF	Superficial layer below a white mat; diffuse flow	Illumina MiSeq 300bp paired-end (2016)	PRJNA785779	SAMN25327505	-
M3	17ROV21CH2P1	Barite chimney	20	none	LCVF	Chimney base; dark grey; diffuse flow	Illumina MiSeq 300bp paired-end (2018)	PRJNA785780	SAMN25350083	-
M4	18ROV28BC3E	Barite chimney	10	none	LCVF	Sediments covered by *Sulfurimonas* mat; 20 cmbsf, dark grey; diffuse flow	Illumina MiSeq 300bp paired-end (2019)	PRJNA785781	SAMN25350084	-
M5	CGB_Flange_2011_3	White smoker	72	none	JMVF	Soria Moria; flange; focused flow	Illumina MiSeq 300bp paired-end (2016)	PRJNA785783	SAMN25350097	Dahle *et al.*, 2015
M34	CGB6_1	Hydrothermal sediments	30	sufite-pulped spruce	JMVF	Bruse Vent Field; in-situ incubators; diffuse flow.	Novaseq S4 150 bp paired-end (2021)	PRJNA801110	SAMN09901170	Stokke *et al.*, 2020
M10	CGB6_2	Hydrothermal sediments	50	sufite-pulped spruce	JMVF	Bruse Vent Field; in-situ incubators; diffuse flow.	Illumina MiSeq 300bp paired-end (2016)	PRJNA801110	SAMN09768205	Stokke *et al.*, 2020
M11	CGB6_3	Hydrothermal sediments	75	sufite-pulped spruce	JMVF	Bruse Vent Field; in-situ incubators; diffuse flow.	Illumina MiSeq 300bp paired-end (2016)	PRJNA801110	SAMN09768207	Stokke *et al.*, 2020

M12	CGB7_1	Hydrothermal sediments	30	salmeal	JMVF	Bruse Vent Field; in-situ incubators; diffuse flow.	Illumina MiSeq 300bp paired-end (2016)	PRJNA801110	SAMN06885931	Stokke *et al.*, 2020
M13	CGB7_2	Hydrothermal sediments	50	salmeal	JMVF	Bruse Vent Field; in-situ incubators; diffuse flow.	Illumina MiSeq 300bp paired-end (2016)	PRJNA801110	SAMN06885933	Stokke *et al.*, 2020
M14	CGB8_1	Hydrothermal sediments	30	unamended sediment	JMVF	Bruse Vent Field; in-situ incubators; diffuse flow.	Illumina MiSeq 300bp paired-end (2016)	PRJNA801110	SAMN09901172	Stokke *et al.*, 2020
M19	CGB7_3	Hydrothermal sediments	75	salmeal	JMVF	Bruse Vent Field; in-situ incubators; diffuse flow.	Novaseq S4 150 bp paired-end (2021)	PRJNA801110	SAMN09901157	Stokke *et al.*, 2020
M20	CGB8_2	Hydrothermal sediments	50	unamended sediment	JMVF	Bruse Vent Field; in-situ incubators; diffuse flow.	Novaseq S4 150 bp paired-end (2021)	PRJNA801110	SAMN09901206	Stokke *et al.*, 2020
M21	CGB8_3	Hydrothermal sediments	75	unamended sediment	JMVF	Bruse Vent Field; in-situ incubators; diffuse flow.	Novaseq S4 150 bp paired-end (2021)	PRJNA801110	SAMN09901209	Stokke *et al.*, 2020

* LCVF: Loki's Castle Vent Fields; JMVF: Jan Mayen Vent Field

Table S2. Detailed results of assembly of metagenomic data from AMOR samples.

sample	no. contigs	total contig length	min. length	avg. length	max. length	platform
M1	166 620	523 823 444	729	3 144	488 446	MiSeq
M2	279 189	496 185 861	186	1 777	281 446	MiSeq
M3	190 141	483 416 503	681	2 542	273 092	MiSeq
M4	67 340	212 151 371	871	3 151	196 444	MiSeq
M5	86 318	234 272 032	840	2 714	173 917	MiSeq
M10	67 679	329 701 234	897	4 872	701 252	MiSeq
M11	62 617	272 737 691	729	4 356	849 685	MiSeq
M12	79 658	324 630 179	803	4 075	492 671	MiSeq
M13	33 210	165 947 731	830	4 997	1 014 593	MiSeq
M14	133 854	408 896 728	753	3 055	449 118	MiSeq
M19	24 609	202 739 854	2 000	8 238	1 104 481	NovaSeq
M20	76 204	547 605 495	2 000	7 186	711 439	NovaSeq
M21	67 392	496 372 703	2 000	7 366	706 890	NovaSeq
M34	70 763	529 148 751	2 000	7 478	836 207	NovaSeq

Table S3. Hallmark plasmid genes identified within the sequences from cluster 2.

gene	start	end	length	annotation_conjscan	annotation_accessions	annotation_description	source
M21_ctg_589898_37	44125	45606	1482	T_virB10	PF03743;COG2948	Type IV secretory pathway, VirB10 components	GeNomad
M21_ctg_589898_38	45606	46502	897	T_virB9	PF03524;K20532;TIGR02781;COG3504	P-type conjugative transfer protein VirB9	GeNomad
M21_ctg_589898_39	46528	47250	723	T_virB8	PF04335;K20531;COG3701;TIGR03781	Type IV secretory pathway, TrbF components	GeNomad
M21_ctg_589898_40	47333	49189	1856	-	-	Type IV secretory pathway	manual annotation
M21_ctg_589898_42	49560	50306	747	T_virB5	COG5314;K20266	Conjugal transfer/entry exclusion protein	GeNomad
M21_ctg_589898_47	52731	55106	2376	virb4	PF19044;PF03135;PF05101;K03199;TIGR00929;COG3451	type IV secretion/conjugal transfer ATPase, VirB4 family	GeNomad
M21_ctg_589898_48	55321	55641	320	-	-	conjugation-related protein	manual annotation
M21_ctg_589898_49	55658	56005	347	-	-	TrbC/VIRB2 family	manual annotation
M21_ctg_589898_51	56255	57217	962	-	-	type II secretion system protein E	manual annotation
M21_ctg_589898_53	58528	59085	558	-	PF10671;PF07283;K20267;COG3417	Toxin co-regulated pilus biosynthesis protein Q; Conjugal transfer protein TrbH	GeNomad
M21_ctg_589898_54	59100	60875	1775	-	-	Type II secretory pathway, component PulD	manual annotation

M21_ctg_589898_56	61651	62820	1169	-	-	Pilin accessory protein (PilO)	manual annotation
M21_ctg_589898_57	62810	64396	1586	-	-	Type II secretion system (T2SS), protein E, N-terminal domain	manual annotation
M21_ctg_589898_58	64383	65549	1166	-	-	Type II secretion system (T2SS) protein	manual annotation
M21_ctg_589898_60	66085	67098	1013	-	-	Type II/IV secretion system protein pilT	manual annotation
M21_ctg_589898_76	79395	80768	1374	-	PF01051;COG5527	Initiator Replication protein	GeNomad
M21_ctg_589898_78	82611	83693	1083	-	PF07506;PF02195;TIGR03454;COG1475;K03497	plasmid partitioning protein RepB	GeNomad
M21_ctg_589898_120	109620	109949	330	-	PF19514;COG4453	MobC-like protein	GeNomad
M21_ctg_589898_123	113049	115877	2829	MOBP1	PF03432	Relaxase/Mobilisation nuclease domain	GeNomad

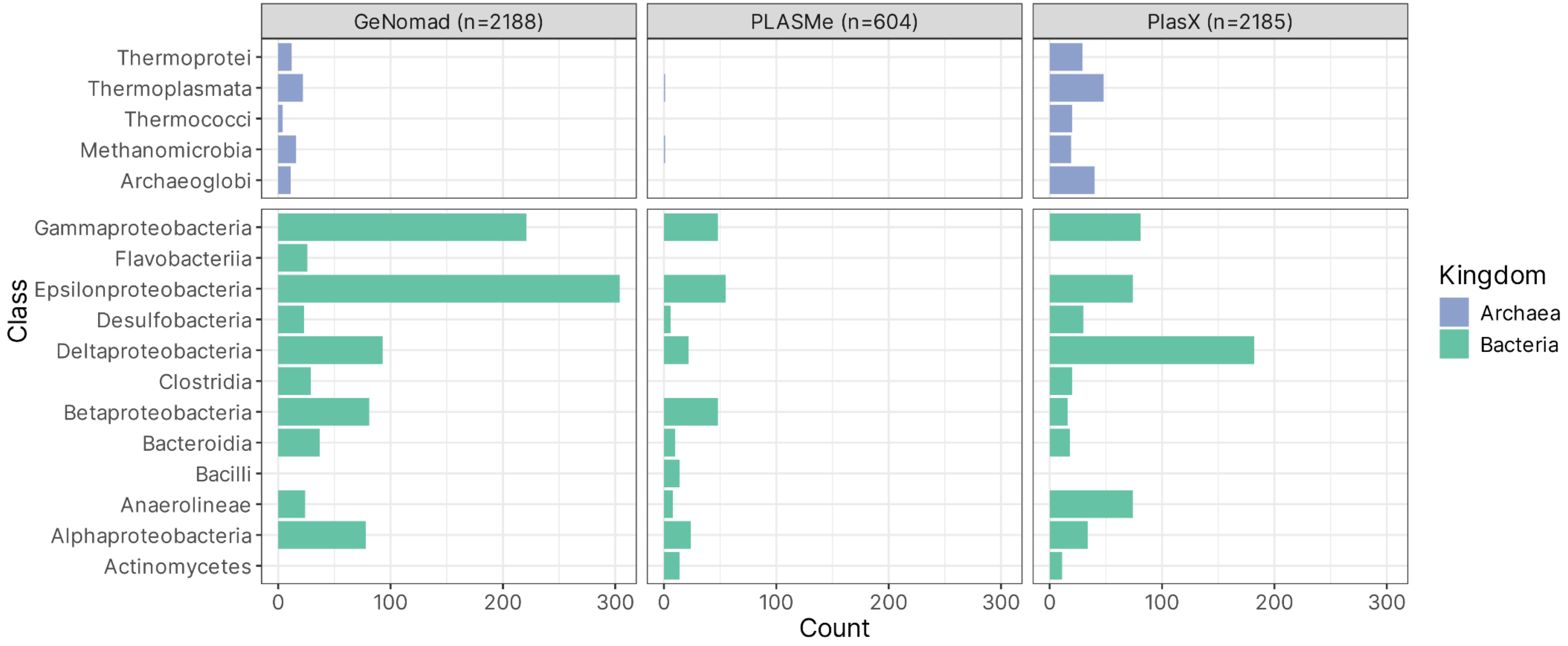

Figure S1. Taxonomic classification of plasmid contigs determined by various bioinformatic tools for each environmental sample.

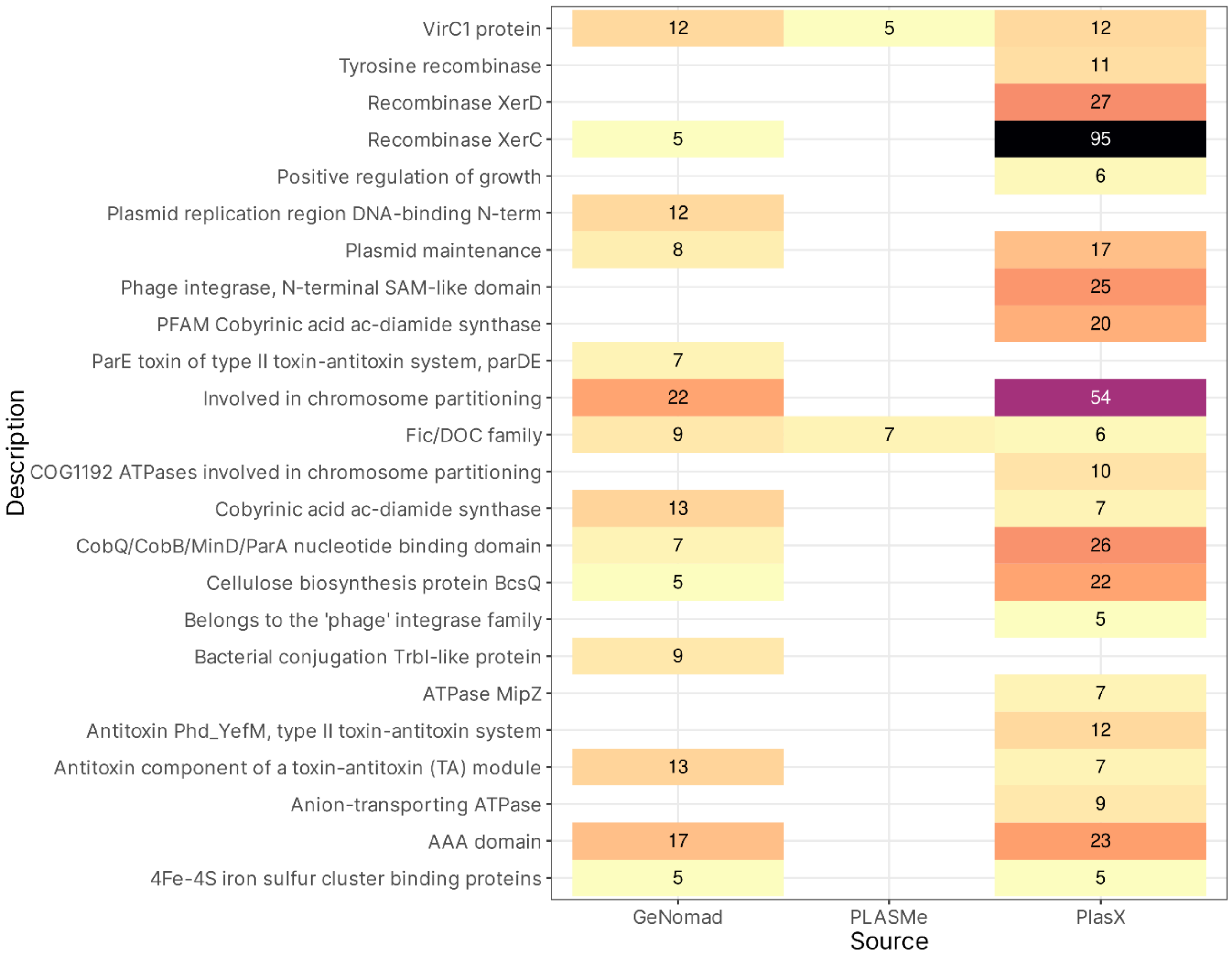

Figure S2. Distribution of annotations of proteins classified as COG category D (cell cycle control, cell division, chromosome partitioning) between plasmid classification tools.

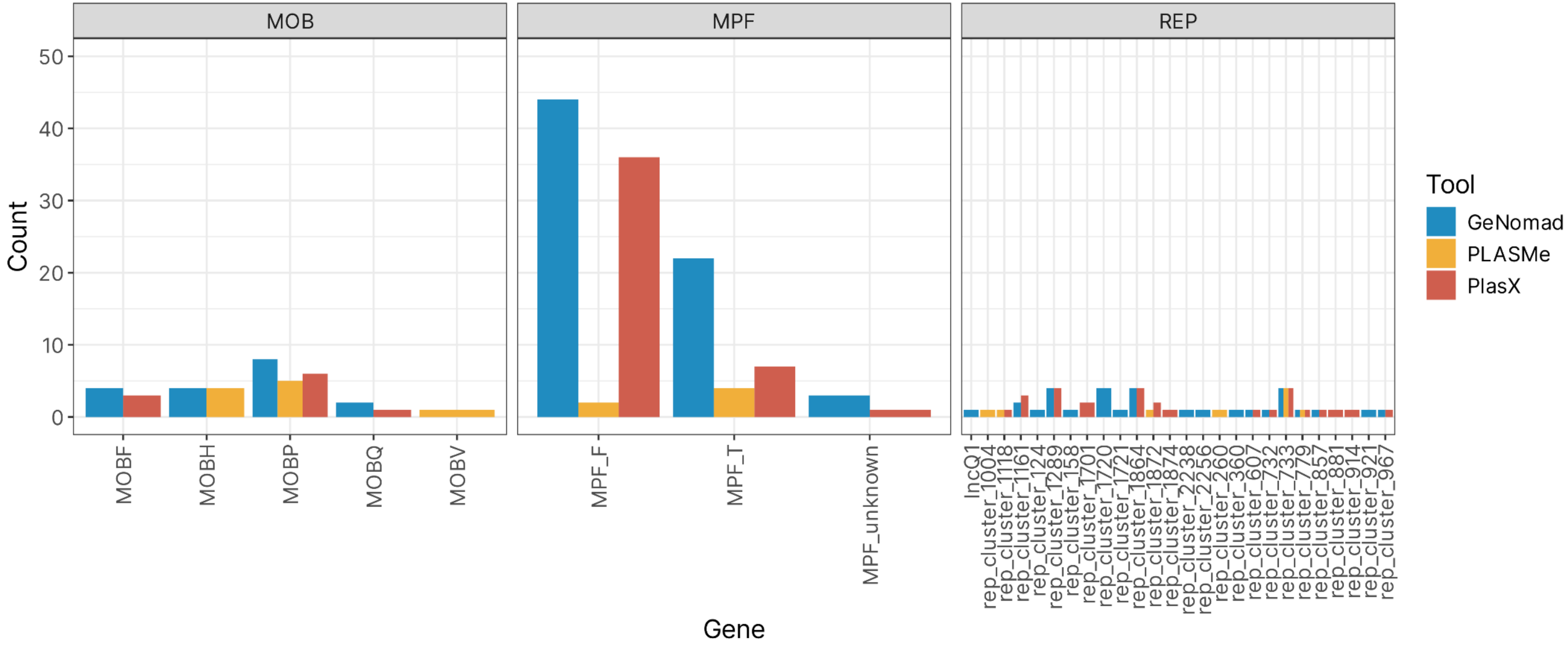

Figure S3. Bar chart showing counts of genes identified by using the MOB, MPF and REP databases in each plasmid contig dataset.

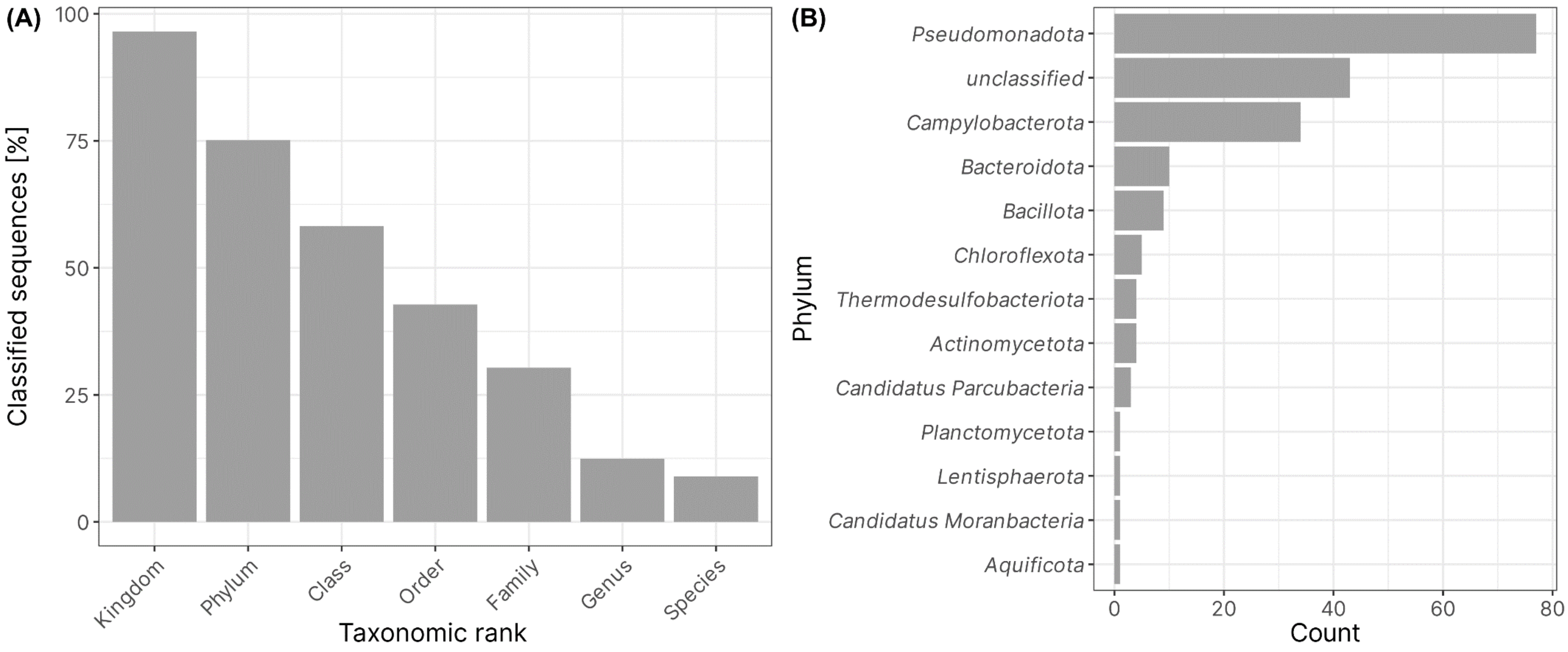

Figure S4. (A) Percentage of classified plasmid contigs within each taxonomic rank (B) Phylum level taxonomy of bacterial plasmid contigs in majority voting dataset

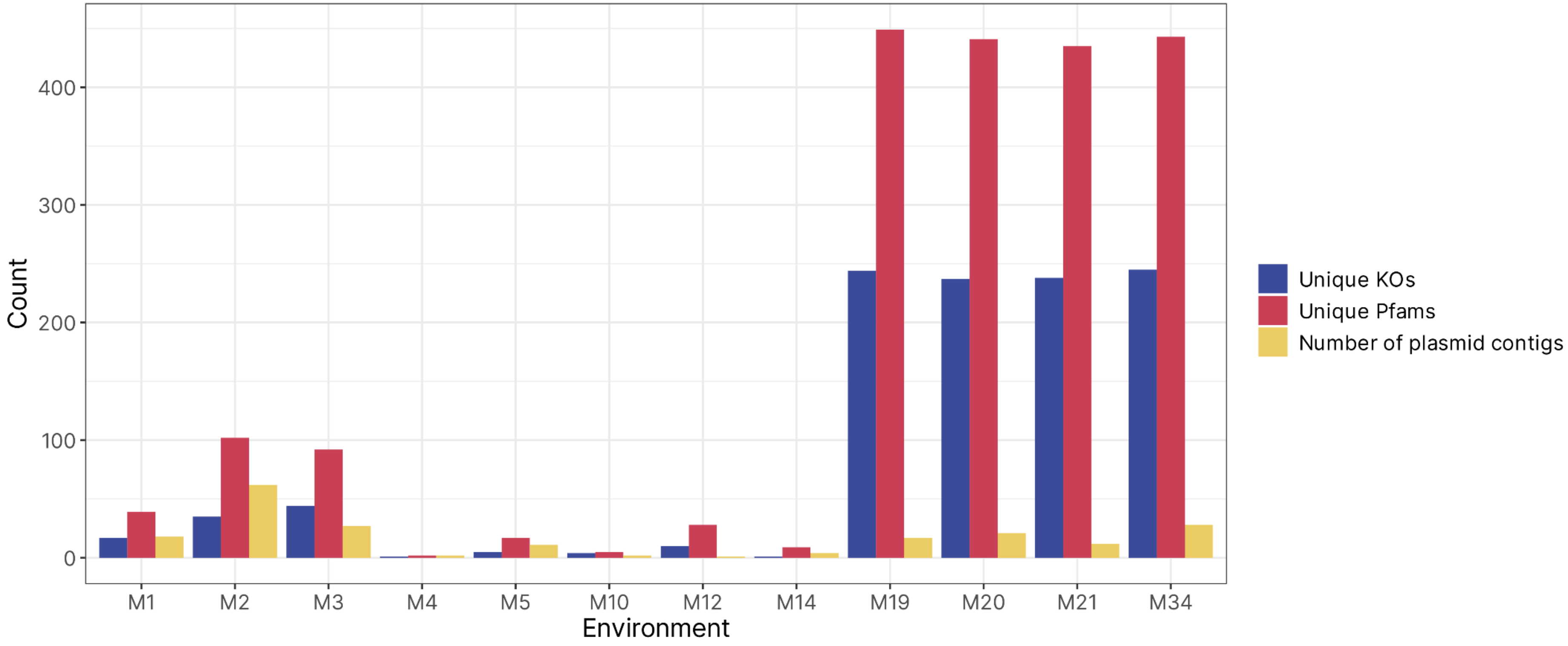

Figure S5. Bar chart comparing number plasmid contigs originating from each environment, as well as number of unique KEGG KO and Pfam IDs assigned to proteins from these contigs.
